# Absence of mtDNA mutations in leukocytes of CADASIL patients

**DOI:** 10.1186/1756-0500-1-16

**Published:** 2008-05-30

**Authors:** Khaled K Abu-Amero, Ali Hellani, Saeed Bohlega

**Affiliations:** 1 Mitochondrial Research Laboratory, Department of Genetics, King Faisal Specialist Hospital and Research Center, Riyadh, Saudi Arabia; 2 PGD laboratory, King Faisal Specialist Hospital and Research Center, Riyadh, Saudi Arabia; 3 Neuroscience department, King Faisal Specialist Hospital and Research Center, Riyadh, Saudi Arabia; 4 King Faisal Specialist Hospital, PO Box 3354, Riyadh, KSA, Saudi Arabia

## Abstract

**Background:**

Ultrastructural and biochemical abnormalities of mitochondria have been reported in skeletal muscle biopsies of CADASIL patients with mutations in the *NOTCH3 *nuclear gene. Additionally, it was proposed that *NOTCH3 *gene mutations may predispose the mitochondrial DNA (mtDNA) to mutations.

**Methods:**

We sequenced the entire mitochondrial genome in five Arab patients affected by CADASIL.

**Results:**

The mean number of mtDNA sequence variants (synonymous and nonsynonymous) in CADASIL patients was not statistically significantly different from that in controls (*p *= 0.378). After excluding haplogroup specific single nucleotide polymorphisms (SNPs) and proved silent polymorphisms, no known or novel pathologic mtDNA mutation(s) could be detected in any patient. Additionally, there was no difference in the prevalence of different mitochondrial haplogroups between patients and controls.

**Conclusion:**

Our study group is too small for any valid conclusion to be made. However, if our observation is confirmed in larger study group, then mtDNA mutations or mitochondrial haplogroups may not be important in the pathogenesis of CADASIL.

## Background

Cerebral autosomal dominant arteriopathy with subcortical infarcts and leukoencephalopathy (CADASIL) (MIM 125310) is characterized by degeneration of vascular smooth muscle cells (VSMC) of nearly all tissues studied to date. The disease is caused by mutations of the *NOTCH3 *gene encoding the transmembrane receptor *NOTCH3*, which is expressed predominantly in VSMC [1]. Electron microscopic examination of muscle and nerve biopsies from CADASIL patients with *NOTCH3 *mutations revealed enlarged mitochondria with needle-like calcium precipitates [2]. A muscle biopsy and brain magnetic resonance spectroscopic imaging, for a CADASIL patient with frameshift deletion in the *NOTCH3 *gene, showed signs of mitochondrial impairment (ultrastructural mitochondrial abnormalities and increased parenchymal brain lactate respectively) [3]. Ultrastructural examination of Fibroblasts and myobalsts from a CADASIL patient revealed mitochondrial aberration and reduced numbers. The patient harbors a *NOTCH3 *mutation and a mitochondrial mutation (5650 G> A) in the tRNA (Ala) gene [4]. Finnila *et al*. assumed that the co-occurrence of the two mutations is not coincidental and that mutations in the *NOTCH3 *gene may predispose the mtDNA to mutations [4]. Sequencing the entire mtDNA coding region in 77 CADASIL patients and 192 matching controls revealed the presence of higher number of polymorphisms among patients in comparison to controls [5]. A significant decrease in the activity of complex I was noted in a muscle biopsy taken from a Spanish CADSAIL patient and histochemical analysis of his affected family members showed the presence of ragged-red fibers with abnormal cytochrome c oxidase staining [6]. Dotti and others looked for common pathogenic mtDNA mutations in DNA extracted from the skeletal muscles of one CADASIL patient, and detected none [3]. Muscle biopsies were not available to us, but given that mitochondrial dysfunctions can now be detected in circulating leukocytes of patients with various mitochondrial disorders [7-10], we sequenced the full mitochondrial genome from DNA extracted from circulating leukocytes of five CADASIL patients in order to search for any pathogenic mtDNA mutation.

## Methods

### Patient's enrollment

**Figure 1:**
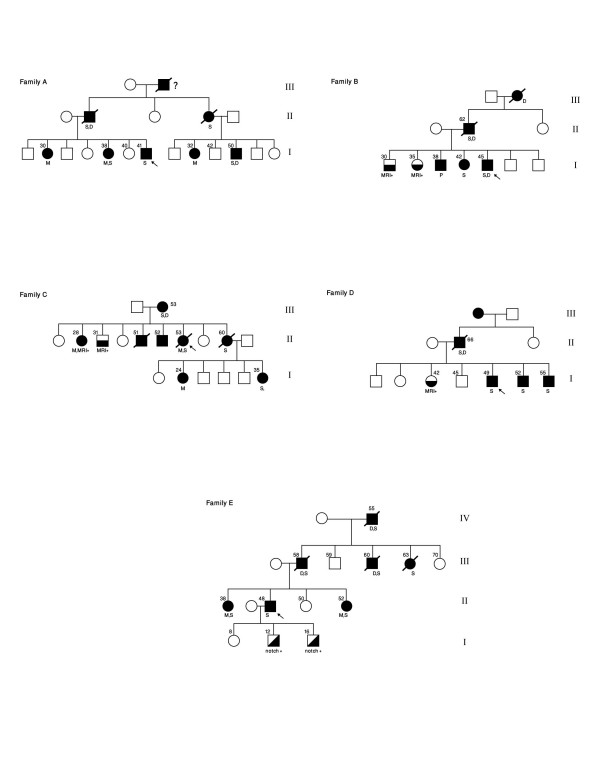
Family Pedigrees.

Requests to identify cases and participants in this study was done through mailing a brief Inclusion Criteria form (see below) for the disease to all members of the Pan Arab Union of Neurological Sciences (PAUNS). Families were included when an index case had both a history of transient ischemic attacks (TIA) or subcortical stroke of unidentified etiology, positive family history of stroke with early death or dementia compatible with autosomal dominant traits, and a cranial MRI scan showing diffuse or focal microangiopathic white matter abnormalities. Five patients (Figure-1) with pure ethnic backgrounds fulfilled the inclusion criteria for CADASIL. Patient (1) and (2) were from Saudi Arabia, patient (3) was from Kuwait, patient (4) was from Sudan and finally, patient (5) was from Yemen.

### Controls enrollment

Control subjects included: 1) 159 Saudi Arabian blood donors at the King Faisal Specialist Hospital and Research Centre who represented the spectrum of the Saudi population; 2) 50 healthy Sudanese individuals; 3) 57 Egyptians; 4) 108 healthy individuals from Yemen and 5) 120 individuals from gulf countries (Oman, Qatar, Kuwait and Bahrain). All Arab subjects in our repository reported no symptomatic metabolic, genetic, ocular or neurological disorders on an extensive questionnaire regarding family history, past medical problems and current health.

### Sample collection and DNA extraction

Ten ml of peripheral blood were collected in EDTA tubes from all participating individuals after obtaining their written informed consent approved by the KFSH-IRB. DNA was extracted from whole blood samples of all CADASIL patients and controls using the PUREGENE DNA isolation kit from Gentra Systems (Minneapolis, USA).

### mtDNA amplification and sequencing

The entire mitochondrial genome was amplified in all patients and controls using primers and amplification conditions described previously [11]. Each successfully amplified fragment was directly sequenced using the same primers used for amplification and the BigDye Terminator V3.1 Cycle Sequencing kit (Applied Biosystems, Foster City, CA). Samples were run on the ABI prism 3100 sequencer (Applied Biosystems).

### Sequence analysis of the mitochondrial DNA genome

The full mitochondrial genome was sequenced. Sequencing results were compared to the corrected Cambridge reference sequence [12]. All fragments were sequenced in both forward and reverse directions at least twice for confirmation of any detected variant. All sequence variants from both CADASIL patients and controls were compared to the Mitomap database [13], the Human Mitochondrial Genome Database [14], GenBank [15], and Medline listed publications. Reported homoplasmic synonymous or non-synonymous (NS; resulting in amino acid change) polymorphisms used predominantly for haplogroup analysis were excluded from further consideration [16].

### Prediction of pathogenicity

Pathologic characteristics of each NS sequence change, in both CADASIL patients and controls, were estimated according to a combination of standard pathogenicity criteria [17] and an evaluation of interspecies conservation using the PolyPhen database [18]. Therefore, a NS nucleotide change was considered potentially pathologic, if it met all of the following criteria, when applicable: 1) it was not found in at least 100 controls of matching ethnicity; 2) it changed a moderately or highly conserved amino acid; 3) it was assessed as possibly or probably pathologic by PolyPhen; 4) it was not established as one of the haplogroup-specific SNPs and 5) It was not reported as an established polymorphism in MITOMAP database, the Human Mitochondrial Genome Database, GenBank, and Medline listed publications.

## Results

Clinical findings and *NOTCH3 *mutation detected in these CADASIL patients have been described previously [19]

Here, we sequenced the full mtDNA genome in five CADASIL patients and 159 ethnicity matching controls. The mean number of sequence changes, relative to the Cambridge reference sequence, detected in CADASIL patients (n = 5) was 18 SD 0.707 and among healthy controls (n = 159) was 17.4 SD 1.51. The difference between the two groups was not statistically significant (*p *= 0.378). All D-loop variants detected in CADASIL patients were also present in controls.

Table 1 details the five non-synonymous mtDNA sequence changes found in CADASIL patients after excluding all synonymous mtDNA changes, established non-synonymous polymorphisms, and non-synonymous mtDNA sequence changes relevant primarily to haplogroup designation. None of these non-synonymous mtDNA sequence changes were novel (not previously reported) and all were not present in ethnicity-matched healthy controls (n = 159). Three of the five non-synonymous sequence variants were transversions and the remaining two were transitions. All were homoplasmic and applying the pathogenicity prediction criteria detailed in methods, none of these non-synonymous sequence changes were potentially pathologic.

**Table 1:** Non-synonymous mtDNA sequence changes detected in CADASIL patients

**Nucleotide substitution**	**Amino Acid substitution**	**Base substitution type**	**Location**	**Hetero-plasmy (%)**	**Interspecies conservation**	**PolyPhen prediction**	**Pathogenicity prediction**
3851 C>G	A182G	Transversion	TM domain of ND1 gene	N/A	Moderate	Benign	Non-pathologic
14113 T>C	F593L	Transition	TM domain of ND5 gene	N/A	Low	Benign	Non-pathologic
14171 A>G	I168T	Transition	TM domain of ND6 gene	N/A	Low	Benign	Non-pathologic
14966 A>C	N74H	Transversion	Outside the functional domain of CYTB gene	N/A	High	Benign	Non-pathologic
15048 G>C	G101A	Transversion	Outside the functional domain of CYTB gene	N/A	Moderate	Benign	Non-pathologic

**Base substitution type – Transversion **= A mutation in which a purine/pyrimidine replaces a pyrimidine/purine base pair or vice versa [G:C > T:A or C:G, or A:T > T:A or C:G]; **Transition **= A mutation in which a purine/pyrimidine base pair is replaced with a base pair in the same purine/pyrimidine relationship [A:T > G:C or C:G > T:A]. **Interspecies conservation **was assessed using PolyPhen [21] which determines interspecies conservation for an altered amino acid by performing alignment with all available amino acid sequences for other species. **Pathogenicity prediction **= A sequence variant was considered potentially pathogenic if it satisfied all the condition detailed in methods. None of above listed non-synonymous sequence changes were found in ethnicity-matched controls (n = 159).

Table 2 details the *NOTCH3 *mutation in each patient and the non-synonymous mtDNA sequence changes detected in each patient. Four CADASIL patients had *NOTCH3 *mutation and one patient from the Sudan had none after sequencing the full *NOTCH3 *gene. None of the CADASIL patients had any potentially pathological mtDNA sequence. Additionally, we classify the CADASIL patients into their respective mitochondrial haplogroup. Each patient belonged to a unique mitochondrial haplogroup (Table 2). All

**Table 2:** Summary of the *NOTCH3 *mutations and mitochondrial abnormalities detected in CADASIL patients

**Patient**	**Ethnicity**	***Notch3*mutation detected**	**NS^+ ^mtDNA sequence changes**	**Mitochondrial haplogroup Of CADASIL patients**	**Percentage● of mitochondrial haplogroup in controls (n = 552)**
1	Saudi	c.406 C> T	14113	M	3.1%
2	Saudi	c.1568 C> T	3851	K	4%
3	Kuwait	c.406 C> T	14171	N	7.4%
4	Sudan	ND	15048	preHV1	17.9%
5	Yemen	c.475 C> T	14966	J	21%

ND = not detected

^+ ^All non-synonymous (NS) mtDNA sequence changes reported above were not detected in 159 matching healthy controls.

● Percentage of Mitochondrial haplogroups among 552 healthy Arabs as previously reported by Abu-Amero et al., 2008 [20]

## Discussion

We have previously described five Arab patients belonging to five unrelated pedigrees with a clinical phenotype typical of CADASIL. *NOTCH3 *mutations were found in four of those and we concluded that CADASIL occurs in Arabs, with clinical phenotype and genotype similar to that in other ethnic groups [19]. As there has been considerable increase in the number of studies demonstrating mitochondrial abnormalities in CADASIL patients, we sequenced the entire mitochondrial genome with aim of detecting established or potentially pathogenic mtDNA sequence changes. One study has shown a higher number of mtDNA-polymorphisms among CADASIL patients in comparison to healthy controls and detected three pathogenic mtDNA mutations in three separate CADASIL pedigrees [5]. However, this was not the case in our study as the number of polymorphisms was similar in both patients and controls. Additionally, we could not detect any pathogenic mtDNA mutation among our study group. The differences in mtDNA-polymorphisms observed in their study could be as a result of differences in haplogroup frequencies between CADASIL patients and controls as was stated [5]. Only 3/77 (3.9%) of their CADASIL pedigrees had pathogenic mtDNA mutations and therefore, this finding cannot be taken as a complete picture. In light of our findings and others [4-6], previous hypothesis that *NOTCH3 *contribution to mtDNA maintenance and that, mutations in *NOTCH3 *increase the frequency of mutations in mtDNA [4] may not be compelling enough. Additionally, our CADSIL patients were assigned into their respective mitochondrial haplogroup. Each patient had a unique haplogroup and those were also present in Arab healthy population [20]. We could not calculate the statistical significant differences in the mitochondrial haplogroup frequencies between controls and patients due to the fact that each patient had a unique haplogroup. Consequently, the number was too small to make a statistical comparison and as a result we could not reach a conclusion whether mitochondrial haplogroup is a risk factor for CADASIL or not. However, if our observation is confirmed in larger study group, then mtDNA mutations or mitochondrial haplogroups may not be important in the pathogenesis of CADASIL. This does not exclude the fact that other mitochondrial abnormalities, such as alteration in mitochondrial respiration or ultra-structure, could be involved.

## Abbreviations

CADASIL: Cerebral autosomal dominant arteriopathy with subcortical infarcts and leukoencephalopathy; EDTA: Ethylene Diamine Tetra-acetic Acid; MRI: Magnetic resonance imaging; mtDNA: Mitochondrial DNA; NS: Non-synonymous; TIA: Transient ischemic attacks.

## Competing interests

The authors declare that they have no competing interests.

## Authors' contributions

KKA was in charge of design and analysis of data, AH performed the technical aspects of the study, PCR and Sequencing, SB was responsible for recruiting patients, thorough clinical evaluation and overall supervision of the study. All authors have read and approved the final version of the manuscript.
